# Supplementary material for: An Alkyne-Mediated SERS Aptasensor for Anti-Interference Ochratoxin A Detection in Real Samples
Source: Foods. 2022 Oct 28;11(21):3407. doi: 10.3390/foods11213407 (PMC9654047; doi:10.3390/foods11213407)
Supplement: Supplementary file 1 [file foods-11-03407-s001.zip › foods-1942479-supplementary.pdf]

An Alkyne-mediated SERS Aptasensor for Anti-interference Ochratioxin A Detection in Real Samples

Hao Wang [1,2], Lu Chen [1,2], Min Li[1,2], Yongxin She[3], Chao Zhu[1,2, *], Mengmeng Yan[1,2, *]

1 Institute of Quality Standard and Testing Technology for Agro-Products, Shandong Academy of Agricultural Sciences, Jinan, 250100, China;

2 Shandong Provincial Key Laboratory Test Technology on Food Quality and Safety, Jinan, 250100, China.

3 Institute of Quality Standard and Testing Technology for Agro-Products, Chinese Academy of Agricultural Science, Beijing 100081, China.

* Corresponding Authors: ndytzhuchao@126.com (C Z); ynky202@163.com (M Y)

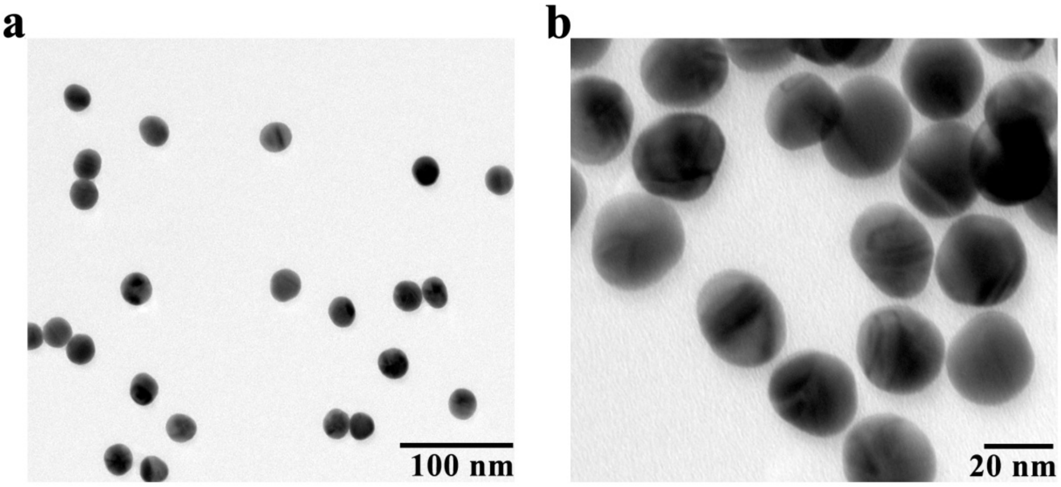

Figure S1. TEM images of **(a)** Au NPs and **(b)** 4-TEAE/Au NPs.

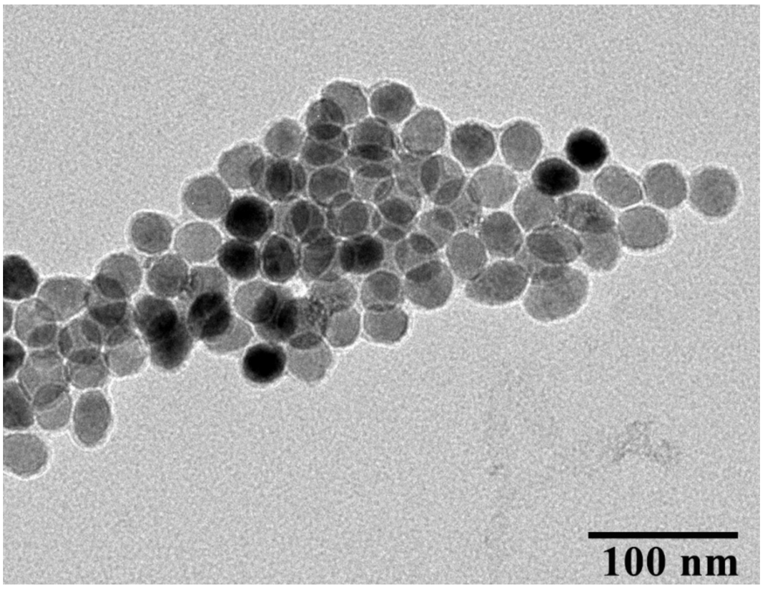

Figure S2. SEM images of Fe_3O_4 NPs.

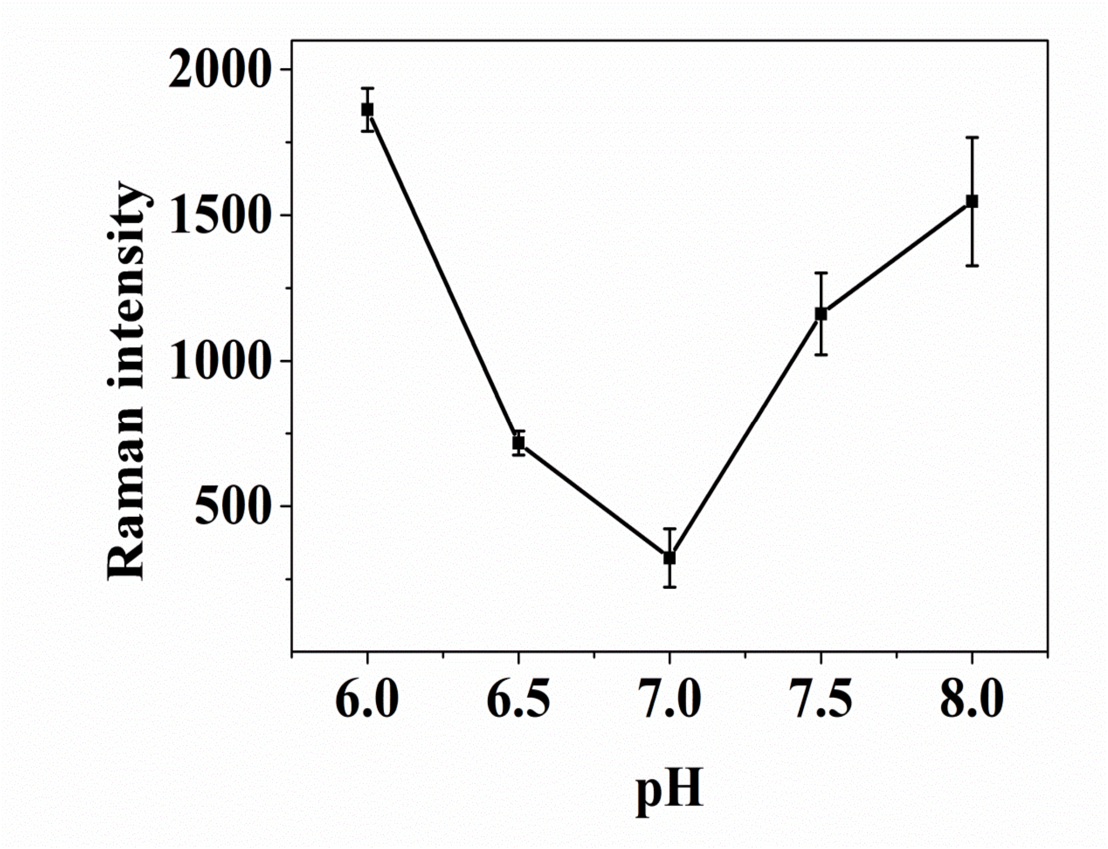

Figure S3. Raman intensity detected at 1998 cm^{-1} for the optimization of pH

Table S1. Comparison of Different Methods for Pesticide Residues Detection

Detection method	Pesticides	Linear range	LODs	Real samples	Ref.
colorimetric	Iprobenfos<br>Edifenphos	1 μM-100 μM	1.67 μM<br>38 nM	Spiked rice	[1]
electrochemical	1-Naphtol	0.1 μM-10 μM	0.27uM	Fruit juices	[2]
fluorescence	Omethoate	0-200 nM	0.22 nM	Cabbage<br>Lake water<br>Apple	[3]
electrochemical	Acetamiprid	5-6000 nM	1 nM	Wastewater<br>Tomato	[4]
fluorescence	Thiamethoxam	10-1000 nM	1.23 nM	Environmental water	[5]
electrochemical	Acetamiprid	0.25-2.0 mM	0.086 mM	Fruit juice	[6]
SERS	OTA	0.1-150ng/mL	0.03ng/mL	soybean, grape and milk	This aptasensor

Reference

1. Kwon, Y.S.; Nguyen, V.T.; Park, J.G.; Gu, M.B. Detection of iprobenfos and edifenphos using a new multi-aptasensor. *Anal Chim Acta*. **2015**, 868:60-66. https://doi.org/10.1016/j.aca.2015.02.020
2. Selvolini, G.; Bajan, I.; Hosu, O.; Cristea, C.; Sandulescu, R.; Marrazza, G. DNA-Based Sensor for the Detection of an Organophosphorus Pesticide: Profenofos. *Sensors*. **2018**, 18. https://doi.org/10.3390/s18072035
3. Zhao, Y.; Wang, Y.; Yang, R.; Zhang, H.; Zhao, Y.; Miao, X.; Lu, L. A zero-background fluorescent aptasensor for ultrasensitive detection of pesticides based on magnetic three-dimensional DNA walker and poly(T) -templated copper nanoparticles. *Sens. Actuators, B*. **2021**, 343. https://doi.org/10.1016/j.snb.2021.130172
4. Fan, L.; Zhao, G.; Shi, H.; Liu, M.; Li, Z. A highly selective electrochemical impedance spectroscopy-based aptasensor for sensitive detection of acetamiprid. *Biosens. Bioelectron.* **2013**, 43:12-18. https://doi.org/10.1016/j.snb.2021.130172
5. Luo, Y.; Jin, Z.; Wang, J.; Ding, P.; Pei, R. The isolation of a DNA aptamer to develop a fluorescent aptasensor for the thiamethoxam pesticide. *Analyst*. **2021**, 146:1986-1995. https://doi.org/10.1016/j.snb.2021.130172
6. Rapini, R.; Cincinelli, A.; Marrazza, G. Acetamiprid multidetection by disposable electrochemical DNA aptasensor. *Talanta*. **2016**, 161:15-21. https://doi.org/10.1016/j.talanta.2016.08.026
